# (Over)crowded house: exploring asylum seekers’ experiences of the COVID-19 pandemic while living at accommodation centers in Sweden

**DOI:** 10.1186/s12889-024-18089-6

**Published:** 2024-02-27

**Authors:** Charlotta van Eggermont Arwidson, Jessica Holmgren, Petter Tinghög, Henrik Eriksson, Kristina Gottberg

**Affiliations:** 1 Department of Neurobiology, Care Sciences and Society, Division of Nursing, Karolinska Institutet, Stockholm, Sweden; 2 Department of Health Sciences, Swedish Red Cross University, Huddinge, Sweden; 3 Department of Health Sciences, University West, Trollhättan, Sweden; 4 School of Health, Care and Social Welfare, Mälardalen University, Eskilstuna, Sweden; 5 Department of Clinical Neuroscience, Division of Psychology, Karolinska Institutet, Solna, Sweden

**Keywords:** Accommodation centers, Asylum seekers, COVID-19 pandemic, Equity, Housing, Qualitative research, Sweden

## Abstract

**Background:**

The COVID-19 pandemic has made visible the scale of health disparities in society, highlighting how the distribution of infection and deaths differs between population subgroups within countries. Asylum seekers represent a potentially vulnerable group; early in the pandemic, concerns were raised about their housing situation, usually involving overcrowded, camp-like accommodations, and the effects of COVID-19 in relation to this. Hence, this study aimed to explore asylum seekers’ experiences of the COVID-19 pandemic while living at accommodation centers.

**Methods:**

In this qualitative study, 14 semi-structured interviews were conducted with asylum seekers at two accommodation centers in Sweden. Participants represented a diverse group of asylum seekers in regard to age, educational background, and gender. Data were analyzed using qualitative content analysis.

**Results:**

Experiences related to COVID-19 were highly dependent on the living situation at the accommodation centers and the experience of feeling unsafe in shared spaces. This was enhanced by the experiences of a challenging mix of COVID-19 messages where different understandings of COVID-19 and related measures existed, together with a feeling of loss of control and safety in shared rooms. Additionally, participants felt more isolated from the outside society and missed prior social activities. Adding to this experience of isolation was an increasing mistrust regarding the authorities’ pandemic response.

**Conclusion:**

This study highlights the importance of understanding the specific challenges and vulnerabilities of asylum seekers at accommodation centers during the pandemic, shaped by their housing situation and legal status. The findings underscore the need for context-specific support, holistic disease prevention approaches, and tailored health communication strategies using diverse formats. Additionally, the findings emphasize the crucial need to identify and mobilize existing community resources in planning and implementing pandemic control measures. Furthermore, the study emphasizes governmental responsibility in providing secure housing, and to address long-term vulnerabilities beyond pandemics.

## Background

From the outbreak of the COVID-19 pandemic in late 2019 to March of 2023, more than 676 million people globally have contracted the virus and more than 6.8 million have died from it [1]. The pandemic caused a global health crisis. In addition, evidence indicates that different groups in society were disproportionately affected by the infection in terms of illness, hospitalizations, and death rates [2, 3]. Data show that socioeconomically vulnerable groups and ethnic minorities, among other things, had higher mortality due to COVID-19 and were over-represented in COVID-19 related hospitalization, thus exposing high levels of health inequity in society [4]. This has highlighted the importance of recognizing the specific needs and protection challenges structurally vulnerable groups in society face, and the necessity to ensure that interventions are based on an understanding of their respective living situations.

Asylum seekers, individuals who have applied for asylum (protection) in another country but have not yet been granted refugee status, were already a vulnerable group in society prior to the COVID-19 pandemic, with higher levels of both physical and mental ill health than among host populations [5–7]. Studies have highlighted risk factors for ill health such as the unpredictable and uncertain asylum process, potentially traumatic experiences from before migration, the vulnerable socioeconomic situation, and a lack of healthcare rights in countries of asylum [8, 9]. Furthermore, the type of housing, such as collective accommodation centers or self-organized housing in the community, has also been highlighted as a determinant of health among asylum seekers [10, 11], with collective institutional accommodations being shown to be related to a higher increase in psychological distress [12]. The discussion on housing form has gained renewed relevance with the COVID-19 pandemic. Early on during the pandemic, researchers and international organizations drew attention to the housing situation of refugees and asylum seekers in refugee camps, or in camp-like conditions at collective accommodation centers. Concerns were raised regarding the overcrowded housing arrangements and poor hygiene situations that were often present in the camps, and a potential higher risk that their residents would contract the disease [13, 14]. So far, there is a limited number of research studies exploring this topic. However, in a study from Greece, the researchers concluded that the risk of contracting COVID-19 was significantly higher among asylum seekers living in camps than among Greece’s host population [15]. Furthermore, there have been reports of large-scale COVID-19 outbreaks in collective accommodation centers for asylum seekers in several European countries, including Germany, Finland, and Italy [16–18]. Researchers have criticized efforts to control COVID-19 for not always taking into account the situation in camp-like settings or not being sufficiently adapted to the characteristics of the centers [19–21]. Other scholars have highlighted discriminatory practices, in which for example mass quarantine measures have been imposed on camps or reception centers exclusively or for longer periods than for other parts of society [18, 22].

In the context of Sweden there are no studies focusing on asylum seekers during COVID-19 specifically, and official population statistics do not include data on asylum seekers. However, what epidemiological studies from Sweden have shown is that foreign-born individuals have been disproportionally affected by the virus in comparison to those who are Swedish-born [2, 23]. Compared to many other countries, Sweden introduced a less restrictive response to the COVID-19 pandemic, mainly relying on recommendations and voluntary measures and with no general lockdown (“lockdown” referring to more comprehensive and widespread restriction of movement and activities imposed by authorities to curb the spread of the virus) [24]. In short, the policy focused on recommendations for physical distancing; avoiding public transport, non-essential travel, and public events; working from home when possible; and self-isolation for those with potential COVID-19 symptoms and people over 70 years of age, with no legal consequences for breaking these rules. The Public Health Agency of Sweden did not issue any specific recommendations regarding protective measures at collective accommodation centers for asylum seekers; the guidelines and action plans regarding efforts to control COVID-19 within these settings were developed by the Swedish Migration Agency during the spring of 2020 (GDs I-002/2020). The guidelines focused mainly on bans on visiting the accommodation centers and the movement of people in risk groups, such as those over 70 or with specific diseases, to their own apartments or rooms.

On arriving in Sweden, asylum seekers can choose their housing form. Around 60% choose to organize housing in the community themselves, most commonly with family or friends [25]. For those who cannot organize their own accommodation, the Swedish Migration Agency offers housing either at larger accommodation centers or in apartment accommodations, with the apartments generally reserved for families with children. At the beginning of 2020, just before the World Health Organization (WHO) declared the outbreak of a global pandemic, there were 40,312 people in Sweden’s asylum system [25]. Of these people, 16,739 lived in state-provided housing facilities (larger accommodation centers or in shared apartment accommodations) at the time of the outbreak.

The pandemic has shed light on a new dimension of living at accommodation centers as it challenges the possibility to adequately protect the residents there. Furthermore, it has highlighted that marginalized groups, such as asylum seekers, might have been disproportionally affected by the virus due to prior vulnerability. Additionally, there seems to be a lack of qualitative research exploring the unique experiences of asylum seekers living at collective accommodation centers during the pandemic. Thus, to sufficiently protect the health and safety of asylum seekers there is an urgency to understand and deepen the knowledge on how the pandemic was experienced by asylum seekers themselves. Therefore, this study seeks to bridge this knowledge gap by giving voice to asylum seekers and thereby hopefully increasing the understanding of their situation. This is important knowledge that could inform the development of adequate support to enhance asylum seekers’ well-being.

### Aim

To explore asylum seekers’ experiences of the COVID-19 pandemic while living at accommodation centers in Sweden.

## Methods and materials

### Design

This study had a qualitative design and is based on individual semi-structured interviews subjected to inductive qualitative content analysis [26–28]. This method was considered valuable for gaining in-depth knowledge on asylum seekers’ experiences. Furthermore, the study is situated in the tradition of constructivism, viewing knowledge and truth as subjective and context-bound rather than objective and fixed. Knowledge is constructed through learning and understanding the subjective meanings people attribute to their everyday experiences within a given context.

### Setting

This study was conducted at two of the largest accommodation centers in Sweden, housing 300 and 550 asylum seekers respectively (Swedish Migration Agency, personal communication, August 12, 2020). Located in rural areas, these centers accommodated asylum seekers from over 50 countries. The residents stayed in shared rooms of two to six people, with families typically staying together. Individuals without family shared rooms with unrelated others, i.e. with people whom they had no prior familial or personal connections. Additionally, residents shared communal kitchens, sanitary facilities, and washrooms with a large number of diverse people. The centers were based mainly on self-catering.

### Participants

The 14 participants were recruited using a purposeful sampling technique. The aim was to recruit a diverse sample in terms of gender, age, and educational background, to ensure that the potential variety of experiences among asylum seekers could be captured. Due to general visiting bans at the centers, the recruitment process was assisted by volunteers from local Non-Governmental Organizations (NGOs) who had access to the centers. Hence, asylum seekers who had participated in activities or were currently involved in support programs offered by these NGOs were individually approached by the volunteers, with an invitation to participate in the study. As indicated in Table 1, the recruited participants showed a wide variation in background characteristics in terms of gender (six women, eight men) and age (22–62 years). They were also from eight different countries of origin – coming from Eritrea, Iraq, Syria, Yemen, Bangladesh, Ethiopia, Sudan, and Afghanistan – and had different educational levels, from no formal education to a university degree. Four participants were married and living with their partner at the center, and three had children living with them. In addition, the participants had been in the asylum process for different lengths of time, from four months to nine years. Some had received their first rejection and were awaiting their appeal decision, while others were still waiting for their first-instance decision.

The interviews were conducted in an early phase of the COVID-19 pandemic, between September 2020 and March 2021 and the so-called second wave, and only two of the participants described having had the experience of being sick in COVID-19 (although not confirmed through testing), both before arriving at the collective accommodation center. The other participants’ experiences were related more to a non-lived experience of an infection that might or might not happen.

**Table 1:** Study participant characteristics

Characteristics	Number of participants
Gender	
Men	8
Women	6
Age	
20–30	5
31–40	3
41–50	3
51–62+	3
Country of origin	
Afghanistan	1
Eritrea	3
Ethiopia	1
Iraq	3
Pakistan	1
Sudan	2
Syria	1
Yemen	2
Level of education	
No formal education	2
Primary	3
Secondary	2
University	6
Marital status	
Married living with partner	4
Married not living with partner	2
Single	7
Widowed	1
Time in the asylum process	
<6 months	1
6 months-1 year	3
1–2 years	1
2–3 years	6
>3 years (max 9 years)	3

### Data collection

Before data collection started, an ethical approval for the study was granted by the Swedish Ethical Review Authority (dnr. 2020 − 00896), and permission to conduct research was obtained from the head of each accommodation center.

This study was part of larger ongoing project focusing on asylum seekers’ experiences, and the data collection was conducted for the purpose of two different studies, one previously described elsewhere [9]. Hence, the interviews were divided into two parts with two different aims in mind. An interview guide was developed beforehand and tested in two pilot interviews, leading to minor adjustments; however, pilot interviews were excluded from the study results.

The semi-structured individual interviews were conducted by the first author (CvEA), a researcher with a background as a trained nurse, encompassing clinical practice in migrant health. There was no pre-existing contact or familiarity between the first author and the participants that could potentially influence the interviews. The interviews were conducted in person, at premises near the centers run by local non-governmental organizations (NGOs), and followed necessary precautions, e.g. physical distancing, and the wearing of face masks. Prior to the interview, participants were provided verbal information in a language of their proficiency—either in English or with the assistance of professional interpreters—regarding the study and their right to withdraw at any time without the need for further explanation. Beforehand, written information about the study was translated into Arabic and Persian, taking into account the most common languages spoken at the centers. Hence, participants with proficiency in these languages were also provided with written information. Written or verbal consent was obtained from all participants. Furthermore, in recognizing the potential for unintentionally raising participants expectations about the asylum procedure, it was emphasized before the interview, that participation in the study would not impact the outcome of their asylum claim. The interview started with questions about personal background, followed by open-ended questions designed to explore the experiences around COVID-19, in accordance with the aim of the study, and included questions such as: How do you experience the COVID-19 pandemic? Can you talk about the support/help you have received related to the pandemic? Follow-up questions were asked to further explore the responses and ensure clarity. Depending on the participants’ preferred language, interviews were conducted either in English between the bilingual Swedish-speaking researcher and bilingual participants (*n* = 5), or in Swedish and the preferred native language, with the assistance of professional interpreters via telephone (*n* = 9). Interpreters from well-established translation agencies were used. The interpreters were provided with instructions before each interview, emphasizing the significance of translating every detail, no matter how minor or seemingly nonsensical. All the interviews were audio-recorded with the consent of the participants, and the recordings were transcribed verbatim by the first author. Following the two different ways of interviewing, with or without an interpreter, the transcripts were in either English or Swedish. Consequently, the analysis involved transcripts in two distinct languages. The full interviews lasted 35–79 min. Throughout the interviews, careful attention was given to any indications of distress, and participants were provided the option to halt the interview, along with information about available nearby support services.

### Data analysis

The transcribed text was analyzed using inductive content analysis according to Graneheim and Lundman [26, 27]. This is not a linear process but instead typically goes back and forth between parts and the whole. The analysis was performed mainly by the first and last author together, starting with reading the transcribed interviews to gain a sense of the whole. This step was followed by dividing the text into meaning units, consisting of sentences or whole paragraphs that were related to each other in content and context. The next step, according to Graneheim and Lundman [27], involves condensation of the meaning units before coding, but as we considered the text to already be condensed in its original form we decided to move directly to coding in order not to jeopardize the analysis [28]. Thus, in the following step we labelled the meaning units with codes. To enable further exploration of the text we started by coding the descriptive manifest content, and from there proceeded to also code the latent, underlying, content. Codes were developed close to the text, considering both subject and context, and with the study aim in mind when labelling the codes. After this step the re-contextualization began, with the sorting of codes from the latent content analysis into subthemes based on their similarities and differences. Examples of the analytical process are given in Table 2. Through a process of reflection and discussion, the first and last author agreed on how to sort the codes. The tentative subthemes and themes were then discussed with all co-authors and revised. Throughout analysis process, the authors continually returned to reading the transcribed interviews in order to never lose sight of the whole.

**Table 2:** Examples from the analysis

Meaning unit	Code	Sub-theme	Theme
“..Yes, contact with Swedes to learn Swedish. I had managed to start a little bit in this school, but due to the Corona pandemic this school is closed right now..”	Loss of possibility to learn Swedish through Swedes due to COVID	Missing prior social activities	Feeling more isolated from the outside society
“And when I came I heard other guy talking to one member of the migration, he was bringing me here, his roommate had coughing and blablabla and they just, No one is sick here, just go back…”	Feeling the Migration Agency is not listening to them	Increasing mistrust of authorities’ support	

## Results

The content analysis resulted in two themes, each with two subthemes: (1) *Feeling unsafe in shared spaces*, with subthemes of *Challenging mix of COVID-19 messages* and *Loss of control and safety in shared rooms*; and (2) *Feeling more isolated from the outside society*, with subthemes of *Missing prior social activities* and *Increasing mistrust of authorities’ support*. An overview of the results is shown in Table 3.

**Table 3:** Overview of the results

Themes	Sub-themes
Feeling unsafe in shared spaces	Challenging mix of COVID-19 messages Loss of control and safety in shared rooms
Feeling more isolated from the outside society	Missing prior social activities Increasing mistrust of authorities’ support.

### Feeling unsafe in shared spaces

The experiences related to COVID-19 were highly dependent on the living situation in shared spaces with unrelated others, for example, of varying ages, educational levels, and countries of origin. This created a mix of COVID-19 messages where participants felt challenged by different understandings about COVID-19 and related protection measures. They also perceived a heightened risk of contracting the virus, but without the possibility to take control over their own protective measures. A situation in which participants felt unsafe.

### Challenging mix of COVID-19 messages

In general, narratives from participants highlighted they had a basic knowledge about COVID-19 and related protective measures, such as practicing physical distancing and regular hand washing. Nevertheless, their narratives also illuminated challenges in understanding and in knowing what applied when it came to safety measures. For instance, apart from the official recommendations on protective measures, participants also expressed alternative beliefs and practices that did not necessarily align with the official guidelines or reflect what was advocated in Sweden, as illustrated by the following quote.

*“We use a lot of garlic and onions which have a good effect against Corona. And if you drink a lot of water and walk a lot, you wouldn’t get infected they say.” (Participant 7)*

In addition, diverse perspectives were expressed regarding the perceived threat of COVID-19 and whether participants felt the need to protect themselves or not.

*“I am trying to stay healthy with like eating good food, the necessary and nothing. I am less in need of protection.” (Participant 9)*

Various conditions within the centers may have influenced this situation. Firstly, participants described the presence of different channels for getting information about this new infection. Social media were described as the main source of information related to COVID-19. Many participants frequently mentioned that their main source of information was websites in their own mother tongue, originating from a broad range of countries. One of the participants experienced this as a source of confusion at the center, as advice and information regarding COVID-19 had mixed backgrounds, creating a situation in which it was difficult to know what really applied in the Swedish context and specifically at the center:

*“There’s been different information from different countries and that’s what has made people confused. Yes, how you perceive and how seriously you take Corona has also been different.” (Participant 2)*

Some participants also expressed a feeling of being vulnerable to misinformation from the media, experiencing that advice and information constantly changed.

*“It was like as if it was the new HIV. Yeah. […] first of all, the news exaggerated it beyond it’s, what they should have done. And then also, the information and the misinformation are beyond your imagination. Because, today they tell you, if you do this you will get the virus, the next day the same thing, if you do the same things, you will not get the virus.” (Participant 8)*

This was experienced to add to the uncertainty as to what to believe, and intensifying feelings of stress and fear.

In addition to using social media, the sharing of information between asylum seekers at the centers was described as an essential way of keeping oneself updated on the COVID-19 situation. This was especially true for those participants who could not access written information, mainly due to low literacy levels, or for those who were not as proficient in accessing information via the Internet. One elderly participant described that she relied on word-of-mouth information between co-residents to get information about the protective measures:

*“So, I live in a camp. I don’t have a TV, I don’t have anything, and this is what I hear from people, that they say like this: There shouldn’t be more than four in a group, you should wear a mask, you should wash with soap, you should use hand sanitizer, that a vaccine seems to be coming. […] We talk among ourselves when we sit and drink coffee outside or something, and then we tell each other like this, that this is how you shouldn’t be.” (Participant 12)*

Although this served as a crucial information source for individuals without access to written information, other narratives highlighted its role in circulating rumors and misconceptions among residents, including rumors about support received and the most effective self-protection measures.

Considering the stories about how the participants accessed information about the pandemic, the official communication from authorities does not seem to have played a central role. It was common among participants to perceive the Migration Agency’s communication about the pandemic as difficult to make use of. It was seen as relying only on written materials taped to the walls or leaflets handed out along with other information about the asylum process. Participants mentioned language differences and illiteracy as barriers to accessing the written information, but also described the perceived limitation that no one was there to explain or answer questions about COVID-19.

The experience of mixed messages around COVID-19 and associated challenges in determining what was reliable information was connected to another dimension that emerged in some of the participants’ stories. It seemed to add a burden to the already overwhelming situation as an asylum seeker, and was described as affecting their receptivity of and motivation for seeking information about COVID-19. In an already difficult situation, characterized by anxiety and hopelessness about the future, participants experienced that the messages around COVID-19 further exhausted them and perhaps also made them indifferent to the pandemic:

*“What does it matter if you read the brochures when the situation isn’t good anyway. You don’t have a good precondition. […] and since we’re younger, we have so much else on our minds. Corona isn’t the first thing to think about today. We think about what will happen when and if we get a residence permit. And it’s like that when you come here, you get up to thirty information sheets that we have to go through and read and then you can’t take it. Many times, you don’t read everything.” (Participant 3)*

Additionally, the challenges arising from the situation of mixed messages on COVID-19 appear to include confusion and insecurity regarding how to act and assess symptoms of illness, as well as low motivation to put oneself forward to be tested for the virus.

### Loss of control and safety in shared rooms

Participants addressed the lack of safety and the loss of control they experienced due to the fact that many of them shared a room with several other people who were unknown to them:

*“At [name of accommodation center], you can’t say that there’s any protection or anything. We have shared toilets, a shared kitchen. There are four people sleeping in the same room, so you aren’t protected there. [..] It would be enough for one of them to be infected and the infection would spread quickly.” (Participant 3)*

The participants also experienced poor hygiene in common areas such as kitchens and toilets as a risk factor for contracting COVID-19. At the same time, they described that their ability to maintain good hygiene was limited in shared spaces where responsibility was shared among many unrelated people with different perceptions of how to maintain good hygiene routines. The picture was complicated by the fact that people with different nationalities, languages, ages, and educational backgrounds lived together. Participants talked about the insecurity of depending on how others behaved and what precautions others took, while having difficulty understanding others’ motivation for acting in certain ways. Some participants described friction between people with different backgrounds arising from such simple things as changed greeting routines due to the recommended COVID-19 precautions, and how others could interpret this as a personal insult. Other participants expressed frustration and criticism in regard to fellow residents for not being considerate and applying protective measures. It was common to link these differences to notions of the tradition, ethnicity, or educational background of others. For instance, some participants explained their roommates’ social behavior, such as the need to see friends and family outside the center, as being linked to their ethnicity and tradition, and therefore also perceived it as difficult to influence and as entailing a loss of control. Overall, this loss of control over one’s personal circumstances created the perception that it was pointless to apply protective measures. Living in small, overcrowded rooms made physical distancing, as well as prescribed isolation in the case of virus symptoms, impossible:

*“And even if you’re protecting yourself, you don’t know what the other people are doing. They go to the city, family. When they come back, they might transmit that thing to you. Yeah, you try to do some things but not so much, you just hope that you don’t get Corona. Because in the kitchen, everywhere, you have to share things and you can’t keep your distance.” (Participant 9)*

This feeling of not being in control of one’s own safety and protection measures in relation to a new contagion also seemed to feed into culturally embedded views of fatalistic or religious beliefs about illness:

*“The only thing one can do is hope for the best […] if I’m going to die by Corona or by accident, if it’s my time then I’ll face it.” (Participant 8)*

Some participants also described the pandemic as a sign from God, and said they relied on faith and prayers for protection.

However, an important difference in the experience of the feeling of having lost control and safety could be noted in those participants who shared rooms only with their family members, their spouse, or children. Firstly, it seemed to give them motivation to protect their family members from getting the virus. It also seemed to give them greater confidence in the possibility to avoid infection through social distancing, which was expressed through descriptions of how they avoided contact with others at the center and of how they engaged in new activities, such as primarily meeting others outdoors.

### Feeling more isolated from the outside society

Although life as an asylum seeker at an accommodation center was one of isolation and inactivity even prior to the COVID-19 pandemic, participants experienced the pandemic adding to this experience. Virtually all of the few activities that existed before the pandemic had closed and were missed by participants. An increasing mistrust regarding the authorities’ pandemic response also added to the experienced social exclusion and isolation from the outside society.

### Missing prior social activities

During the pandemic and the related restrictions implemented to minimize the spread of the virus, life at the accommodation centers became even more monotonous than it was before. Participants described that all activities were stopped, volunteers from civil society no longer organized language classes or other activities, and the participants had less contact with the outer society, further increasing their experienced isolation. Even though they had experienced immobility and isolation before the pandemic, some participants stressed that the pandemic had worsened their situation, expressing that they missed engaging in social activities. There were stories about how losing access to people outside the center removed their possibility to learn about Swedish society, learn Swedish, or feel they were part of the society at large.

*“There is not a lot going on. I would have gone to xxx [name of city] and been part of the society. There might have been so many occasions, lots of gatherings, many things to do, but these things are not” (Participant 11)*

Above all, many participants expressed concerns about how inactivity and fewer social contacts could affect their health, especially their mental health. For example, a young asylum seeker explained that he missed going to the gym, which he associated with reduced stress:

*“It’s just like I forget things for a moment. That’s helping me. You know some people, when they’re sad, they drink and they smoke to forget things for the moment. So, for me, gym was like that.” (Participant 9)*

Another participant expressed intensified feelings of anxiety and worries thinking about what disrupted social activities and social contact could mean for his mental health. He had experience of being depressed during an earlier period of waiting in the asylum process, which also led him to state that he was more worried about being depressed again than contracting the virus. Several participants described social relationships as essential for mental health reasons, and sometimes felt they were the only strategy available to feel better about the situation. Therefore, physical distancing also seemed to be experienced as counterproductive in the overall health picture:

*“I feel very bad, I get very stressed especially when I do nothing, and I have to meet new people or people in general. […] I was quite worried after this with, yes if you were to isolate yourself because of Corona. It affected me quite negatively because it would limit my freedom a little. [..] It feels too hard. We would get depressed.” (Participant 7)*

The cancellation of social activities seemed to reinforce the ongoing feeling of uncertainty and hopelessness associated with being an asylum seeker, a feeling that was framed by several participants as the central factor affecting their health and well-being, and sometimes seen as far more damaging than the impacts of the pandemic itself.

Participants also voiced feelings of contradiction when it came to the recommendation of social distancing and the closing down of activities at the center. Considering their situation in overcrowded facilities, the closing of activities seemed to be based on the best interests of civil society organizations or authorities rather than those of asylum seekers:

*“But because of the pandemic we can’t gather around here. But basically, what we have here is gathering. Can you see the irony in our being allowed to get together in our kitchen but not being allowed to gather here?” (Participant 8)*

Participants experienced they were still forced by the spatial conditions at the center to meet other people, but instead of meeting in facilities outside their living spaces they were now limited to gathering in shared spaces such as kitchens.

### Increasing mistrust of authorities’ support

In relation to the pandemic and protective measures, the participants gave different pictures of how they experienced the support they received, suggesting that it was dependent on the individual’s confidence in their own ability and expectations in regard to the support. Some participants described the support as sufficient but rather invisible, except for the written information taped to walls and doors at the centers. They believed that if one needed the Migration Agency and asked for help, the Migration Agency would do their best to support them. Others were more critical, and their experiences seemed to be more embedded in a general feeling of mistrust of the Migration Agency and a perception of being vulnerable as an asylum seeker. These participants seemed to perceive the written information taped to doors and walls as a maintained distance on the part of the authorities, an absence of physical presence, and a sign of not caring enough for their well-being:

*“We don’t get any information from immigration other than these posters and stickers on the doors […] No one from immigration is talking to any of us here. If you want something you go there, but they don’t say anything.” (Participant 1)*

The lack of outreach support, with physically present people to talk to, had a connotation of being left on their own. Additionally, it seemed to have fueled a distrust regarding the Migration Agency’s ability to understand the asylum seekers’ needs and perhaps above all regarding their willingness to provide tailored support.

Several participants talked about the support they received as insufficient or, when discussing special efforts put in place by the Migration Agency to prevent the spread of infection, as a failure. Additionally, the narratives illuminated that stories and rumors circulated within the centers regarding the conducted efforts, contributing to heightened mistrust. Based on one of these stories, one participant described a “quarantine house” at one of the centers – where people with symptoms of infection could stay – as thoughtless, as it did not account for the fact that sick people still needed to eat:

*“My thought is, they’ve put these people who think they have Corona in those rooms in that house. But what do they think? How should they eat and so on. I don’t understand what they’re thinking. There’s no one to help them either. They have to go shopping and do other things. It doesn’t feel like it was a good idea to just send them into that house. How are they going to go about it? [..] I don’t feel they [The Migration Agency] care about us that way. That we’re not worth very much, that they kind of do something just to have done it.” (Participant 4)*

These stories also seemed to fuel a feeling of not being seen as someone worthy of help or that the special efforts were not based on their best interests, further increasing their distrust of Swedish authorities.

Some participants brought up their own ideas about enforcing protection against the virus that they would have liked to see put in place, like a quarantine house for newcomers to make sure they showed no signs of sickness before being placed in rooms with several unrelated others. However, at the same time they lamented the lack of channels for putting forward these ideas to the authorities, implying that they had not been invited to be involved in developing efforts regarding protective measures important to their daily life.

*“I would even say this to the migration agency, I didn’t, because there is no such platform to tell them that this could be good to do..” (Participant 8)*

In some cases, participants also expressed an increased awareness of their vulnerability as asylum seekers, especially in relation to healthcare, fearing they would not get the care they needed if they fell ill due to being an asylum seeker. This experience of feeling vulnerable as an asylum seeker and at the same time not trusting that the support they received was based on real care for their situation was also notable in statements concerning what participants perceived as needs in terms of support related to the pandemic. Most of them stated that they considered a residence permit to be the best help, which not only reveals that some consider the hardships encountered during the asylum process to be more pressing than worries about getting infected, but also contains the aspect of the vulnerability of having to trust and be dependent on someone else for protection in a pandemic situation.

## Discussion

This study aimed to explore asylum seekers’ experiences of the COVID-19 pandemic while living at collective accommodation centers in Sweden. The findings highlight that the experiences were intimately associated with the housing situation and with how the pandemic was handled by the authorities. Asylum seekers at accommodation centers felt unsafe with the housing arrangements and experienced a loss of control in shared spaces. They were confronted with mixed COVID-19 messages, where they felt challenged by different understandings of the virus and associated protection measures. Moreover, they experienced heightened isolation, and increased inactivity due to disrupted social activities. In addition, the experience of isolation was reinforced by a growing lack of trust in the authorities. Their experiences also show that the authorities appeared to rely on written information in public spaces; beyond this, the asylum seekers perceived that they were largely left on their own. They had to acquire an understanding of the situation mainly based on their own capacities and ability to maneuver within their situation during the pandemic.

It is not an underestimation to say that the COVID-19 pandemic was a challenging time for the entire community. However, what our study reveals is that the situation for asylum seekers at accommodation centers was unique and added another dimension to the experience. The findings underline the importance of bringing to light the experiences of asylum seekers to comprehensively grasp the impact of the COVID-19 pandemic in society. Already prior to the pandemic, a large body of research had described asylum seekers’ situation as precarious and as potentially harmful to their health, both physical and mental [29, 30]. Scholars have depicted it as a life in limbo [31] or as living a frozen life [9]. Thus, the effects of the COVID-19 pandemic added to already existing challenges and can be understood based on the concept of *a crisis within a crisis*, which has been aptly used to describe the situation of other vulnerable refugee groups during the pandemic [32]. *A crisis within a crisis* signifies that existing and prior vulnerabilities were exacerbated while the pandemic generated new forms of vulnerability, which we believe is illustrated by our study of asylum seekers in accommodation centers in Sweden. Our results are in line with findings from other studies conducted during the pandemic, which have shown that asylum seekers not only faced a higher risk of COVID-19 infection due to difficult living conditions [15] but also risked an exacerbation of pre-existing mental health issues caused by increased stress, anxiety, and uncertainty [33]. In terms of the exacerbation of pre-existing mental health issues, our study suggests that the disruption of social activities was as contributing factor. Participants described this as a loss of a resource for managing their mental health. This is in line with a growing body of research on enablers of psychological wellbeing for asylum seekers, highlighting social support as a crucial protective factor for mental health, social support including engagement in social activities as well as emotional support [34]. Moreover, while the study populations may not align entirely with ours, research studies centered on broader groups of refugees or elderly women have underscored the crucial role of social support in promoting mental health during the pandemic [35, 36]. Our results point to that civil society organizations had provided vital social support prior to the pandemic. Their closure left asylum seekers with few possibilities to break the isolation and monotony within the centers. Furthermore, our findings indicate that when the civil society organizations left, it made visible that the authorities might have been ill-prepared to provide support to asylum seekers on their own, emphasizing the role of civil society in supporting asylum seekers. This is a relatively underexplored research area, yet existing studies substantiate the theory that NGOs play a crucial role in filling gaps for asylum seekers [37, 38]. This extends to providing support not only in terms of social activities but also in terms of material assistance to alleviate poverty. Taken together, this implies that interventions to promote social support should be part of pandemic response. Other studies conducted during the pandemic, which focused on asylum seekers at collective centers, have emphasized that by failing to consider pre-existing structural vulnerabilities in developing strategies to control the pandemic, national and local institutions were unable to provide adequate and effective protection that guaranteed the well-being and safety of people living at reception centers [18]. This underlines the importance of understanding the experiences of asylum seekers at the intersection of two crises – or, as stated earlier, in a *crisis within a crisis* – in order to adequately meet their needs.

In addition to the importance of social support, our study draws attention to other important factors in relation to what needs to be emphasized in efforts to promote the health and safety of asylum seekers in a pandemic. The participants’ narratives highlight the significance of trust and participation in prevention measures. This aligns with what scholars studying both earlier pandemics and the current one have concluded: that applying core principles of health promotion in pandemic control efforts is crucial [39, 40]. While it is necessary to protect people from physical disease during a pandemic, it is equally important to consider the bigger picture of health, including mental health and overall well-being and the social factors underlying it, to avoid the further marginalization of vulnerable groups and to empower people to act for their own health. When it comes to trust, other studies have emphasized this as a crucial factor in pandemic control efforts [39, 41]. In an environment of trust, people tend to have more confidence in authorities’ recommendations and to adopt preventative behavior to a greater extent. It may also influence people’s intention to be tested or vaccinated. Furthermore, recent studies conducted during the COVID-19 pandemic suggest that low trust in, for example, healthcare systems increased psychological distress among vulnerable groups in society, which also highlights the importance of trust for people’s overall well-being [42]. Our findings illustrate how factors like the perceived gap between official recommendations, for instance regarding physical distancing and good hygiene, and actual living conditions contributed to increased feelings of mistrust. Narratives also show that adding to this was the experience that the control or protection efforts implemented by authorities were perceived as ill-conceived and as not having been implemented with the asylum seekers’ best interests in mind, further decreasing their trust in authorities and the perceived gap in relation to the outside society. A similar finding has been described in an ethnographic study researching the effects of the COVID-19 pandemic in different refugee camp settings [43]. In the case of a German accommodation center, the top-down camp management and implementation of protective measures was interpreted as fostering the experiences of mistrust and conflict during the pandemic. In addition, researchers have described trustworthy relationships as countering the spread of misconceptions and rumors during a pandemic [41]. Our results indicate that reduced trust in authorities during the pandemic may have increased worries and anxiety among asylum seekers, with substantial risk of an exacerbation of their already precarious mental health situation.

In regard to building trust in a pandemic, several studies have highlighted the importance of transparency, as well as clear communication grounded in an understanding of the individuals one wish to communicate with, considering their actual needs and available resources [40, 41]. In relation to this there are also studies that highlight the importance of paying attention to the role of health literacy [44]. Health literacy can be described as a person’s ability to find, understand, communicate, judge, and use health information to maintain their health [45]. This means that helpful information needs to be not only available but also understood, accepted, and possible to apply. It also means that it is not enough to focus on the content of information; one must also consider *how* the information is disseminated. The general abundance of information about the COVID-19 pandemic in society, both true and false, has been described as an infodemic, highlighting that the information flow was challenging for the entire community [46]. However, what our findings point to is that living at an accommodation center added another dimension to these challenges. The diversity of information sources from all over the world, unfamiliarity with official Swedish channels, isolation from the external society, language barriers, illiteracy, and a great heterogeneity of people with different resources, challenges, and needs created a unique and complex situation within the boundaries of the accommodation centers. These findings suggest that to enhance trust and transparency, and develop clear communication, there is a need for health communication strategies that build on an understanding of, and are adapted to, the unique experiences within the accommodation centers. There is also a need for different formats for communication, for example videos and photos as a complement to written information, in a wide range of relevant languages [43]. Furthermore, it is important that messages focus on what people can do in practice to keep from being infected, based on actual circumstances at accommodation centers. In addition, when it comes to the importance of building an environment of trust, scholars have suggested that trusting relationships need to have been built prior to any pandemic [41]. Trust is highly influenced by previous experiences and is generally born out of close and regular contact with populations in vulnerable situations that resonate with the actual life circumstances of the population in question [47]. Thus, our findings emphasize that being prepared for future pandemics requires engagement with marginalized populations already before the pandemic emerges.

Another finding in our study suggests that it is possible that in a situation in which one is already overwhelmed by uncertainty and hopelessness in the asylum process, it may be difficult to process new information. Furthermore, participants described the worries and hopelessness in the asylum process as a barrier to engaging in information on COVID-19. However, more research is needed on how this affects communication or serves as a barrier to receiving information in the context of pandemics, as well as how this can be mitigated in targeted support.

The study also calls attention to the importance of community engagement. Our findings show that asylum seekers themselves requested opportunities to bring forth proposals and opinions about the pandemic response at the centers but perceived that channels for this were lacking. Studies from previous pandemics have shown that community engagement can build trust as well as make a substantial difference both in health outcomes and in strengthening the capacity to handle the pandemic impact on local levels [39]. Furthermore, scholars have concluded that a response to a pandemic outbreak may be more successful if local community knowledge and experiences are validated and combined with top-down managed support and expert knowledge. This could provide valuable information on adjustments to local conditions as well as create a sense of ownership at the grassroots level, which might also be important for enabling people to increase their control over their health, and could create an environment of trust, all of which are beneficial to people’s health [40]. Similar result has been shown in a German study on refugee women in reception centers, where the authors conclude that engaging the community in planning and designing these settings can moderate social conflict and the adaptation to the physical surrounding [48]. In another study from Sweden, which centered on asylum seekers at accommodation centers, it was observed that despite facing challenges, asylum seekers at accommodation centers developed caregiving networks and peer support systems to care for each other [9]. In conjunction with the findings from the current study, these studies reinforce the importance of investigating existing resources within the community that could be supported or hold the potential for collaboration.

Finally, our study underlines that structural factors such as housing have a major impact on people’s health. Disease does not occur independent of social and ecological factors; rather, these factors shape the disease process as well as people’s subjective experiences [49]. Similar findings have been made in a recent ethnographic study, whose authors concluded that the housing situation had a great impact on the experiences of asylum seekers [49]. Whereas those in individual apartments suffered from loneliness and a loss of social contact, those living at collective reception centers suffered from living in close quarters with unrelated others and the inherent loss of privacy and intimacy. However, to nuance the picture, housing can also be associated with opportunities, such as social contact. For example, our findings indicate that participants with low literacy levels or a lack of digital skills (digital illiteracy) may have benefited from caring fellow residents giving them access to verbal information at the accommodation centers. Based on this it might be argued that it is necessary to have a systems approach, which considers the complex interplay between individual, community, and policy factors in analyzing needs and developing interventions [9, 40]. To promote a supportive environment that sustains the health and safety of asylum seekers during a pandemic engages not only the individual level but the community and policy levels as well. However, it cannot be overlooked that asylum seekers exist within a politized migration system and that housing for them is essentially a political decision. On that note, there are researchers who suggest that access to resources within the asylum support system, such as housing, are used as tools to deter people from seeking asylum in the country, referred to as deterrent approaches to asylum support [10]. This might suggest that at the intersection between politically imposed structural conditions in the asylum system and public health interests there exist tensions that must be overcome in order to create a supportive environment that protects asylum seekers’ health and safety. Nonetheless, our study indicates that policy changes are needed to improve asylum seekers’ conditions for health and to prevent the inequity in health that the pandemic has made visible. Early on during the COVID-19 pandemic, international organizations such as the European Centre for Disease Prevention and Control (ECDC) and the Inter-Agency Standing Committee (IASC) developed guidelines that warned about camp-like settings, such as reception/accommodation centers, being hot spots for infection and recommended a range of efforts to ensure the safety and health of those living there. One example of these guidelines is the recommendation that when physical distancing and other containment measures cannot be implemented at reception centers, measures to decongest and evacuate residents should be considered [50]. In accordance with these recommendations, the policy implications of this study also entail that authorities should strive to provide proper housing, in which decongestion needs to be considered as a response to pandemic outbreaks.

There are several implications from these findings. Firstly, the study highlights the urgent need to identify, and design trusted services and support in the context of a pandemic, including communication that counterbalances mistrust and isolation. Clear and consistent public health messages need to be developed based on local circumstances and consider different levels of health literacy within the community. This also calls for future research with the potential to inform the development of communication strategies that builds trust in a pandemic. In addition, the pandemic brought significant changes in social support for asylum seekers and acknowledging social support’s crucial role as a protective factor for mental health, strategies to promote health for asylum seekers during pandemics also need to include approaches to enhance social support. This might involve developing social support through online platforms. Furthermore, public health institutions need to engage with and include marginalized groups in planning and implementing control measures as well as in developing preparedness plans for future emergencies. Diminishing the impact of the next pandemic, should there be one, requires engagement with vulnerable populations prior to the pandemic that builds trustworthy relationships, which are essential in preventative efforts in pandemic response. The findings also serve as a call for healthy public policies that uphold asylum seekers’ rights to health and safety. The pandemic calls for policy and governance led by a sincere intention to work for equity and health for all. This is particularly important as the pandemic also taught us that we are all interconnected when it comes to health and well-being. The well-being of each depends on the health of all. Lastly, as the concept of *crisis in a crisis* suggest, it is crucial to acknowledge that the pre-existing vulnerability of asylum seekers heightened the impact of the pandemic on them. Addressing the issue of reducing asylum seekers’ vulnerability to future pandemics necessitates an improvement in their life circumstances beyond pandemic periods.

### Strengths and limitations

To the best of our knowledge, this study is one of the first to explore the experiences of asylum seekers at accommodation centers in the context of the COVID-19 pandemic. It thereby contributes to our understanding of the living conditions of one of the most marginalized groups in society. This is key to informing preparedness plans to meet their needs should there be a new pandemic, and can be built upon in future studies. However, this study is not without its limitations. It presents the experiences of a relatively small and heterogenous study sample of asylum seekers in Sweden. Therefore, it might limit the capacity to fully capture the extent of experiences of asylum seekers residing in accommodation centers during the COVID-19 pandemic. Additionally, the limited sample size precluded the exploration of variations in findings based on factors such as gender, country of origin, age, or length of stay in Sweden. Undoubtedly, these are factors that shape the experiences in diverse ways. Furthermore, as the study context was determined by the local and Swedish migration policies and COVID-19 measures, its transferability to other contexts might be limited. In addition, the research was conducted during a specific period of time, the so-called second wave of the pandemic, and in light of how the pandemic evolved the findings might be limited to representing a certain phase of the pandemic’s development. Moreover, the recruitment of study participants was limited to individuals engaging with the support activities offered by the collaborating NGOs, which may have resulted in the sample representing a particular group of asylum seekers and may have limited the findings. Furthermore, we used professional interpreting services, which enabled us to overcome language barriers. However, with interpreters there is always a risk of misinterpretation and loss of information, which might have had an impact on the analysis.

## Conclusion

This study has described how asylum seekers experienced the COVID-19 pandemic while living at collective accommodation centers. Based on the findings, we can conclude that living at collective accommodation centers during the pandemic added another dimension to the experiences. Our study draws attention to the fact that asylum seekers at accommodation centers felt unsafe and did not trust pandemic control measures to be implemented with their best interest in mind. This was reinforced by the fact that they did not perceive universal strategies for disease prevention to be applicable or to sufficiently mitigate the impact of the COVID-19 pandemic. This have highlighted the importance of contextualizing support and of taking a more holistic approach to disease prevention, including incorporating support to enhance social support. It also emphasizes that effective health communication strategies should be tailored to the unique experiences of asylum seekers, utilizing diverse formats such as videos and photos alongside written information in various languages. Additionally, authorities should strive to counterbalance mistrust and feelings of isolation through transparent and inclusive communication. Furthermore, the findings call attention to the importance of identifying and mobilizing existing resources and structures within the community in the planning and implementation of interventions to contain the spread of a virus. This could offer vital insights for adapting to local conditions, cultivating an environment of trust, enhancing individuals’ control over their situation, all contributing to improved well-being of asylum seekers during the pandemic. Finally, the study highlights the responsibility of governmental agencies to provide proper housing in order to ensure the safety and health of asylum seekers. This includes measures to decongest and evacuate residents if needed. It also encompasses initiatives to systematically alleviate the vulnerable circumstances of asylum seekers over the long term, extending beyond pandemic situations.

## Data Availability

To protect the participants’ privacy, the dataset generated during the current study is not publicly available. However, it is available from the corresponding author on reasonable request.
